# Deep learning auto-segmentation on multi-sequence magnetic resonance images for upper abdominal organs

**DOI:** 10.3389/fonc.2023.1209558

**Published:** 2023-07-06

**Authors:** Asma Amjad, Jiaofeng Xu, Dan Thill, Ying Zhang, Jie Ding, Eric Paulson, William Hall, Beth A. Erickson, X. Allen Li

**Affiliations:** ^1^ Department of Radiation Oncology, Medical College of Wisconsin, Milwaukee, WI, United States; ^2^ Elekta Inc., ST. Charles, MO, United States

**Keywords:** auto-segmentation, multi-sequence MRI, abdominal organs, deep learning, MRI-guided RT

## Abstract

**Introduction:**

Multi-sequence multi-parameter MRIs are often used to define targets and/or organs at risk (OAR) in radiation therapy (RT) planning. Deep learning has so far focused on developing auto-segmentation models based on a single MRI sequence. The purpose of this work is to develop a multi-sequence deep learning based auto-segmentation (mS-DLAS) based on multi-sequence abdominal MRIs.

**Materials and methods:**

Using a previously developed 3DResUnet network, a mS-DLAS model using 4 T1 and T2 weighted MRI acquired during routine RT simulation for 71 cases with abdominal tumors was trained and tested. Strategies including data pre-processing, Z-normalization approach, and data augmentation were employed. Additional 2 sequence specific T1 weighted (T1-M) and T2 weighted (T2-M) models were trained to evaluate performance of sequence-specific DLAS. Performance of all models was quantitatively evaluated using 6 surface and volumetric accuracy metrics.

**Results:**

The developed DLAS models were able to generate reasonable contours of 12 upper abdomen organs within 21 seconds for each testing case. The 3D average values of dice similarity coefficient (DSC), mean distance to agreement (MDA mm), 95 percentile Hausdorff distance (HD95% mm), percent volume difference (PVD), surface DSC (sDSC), and relative added path length (rAPL mm/cc) over all organs were 0.87, 1.79, 7.43, -8.95, 0.82, and 12.25, respectively, for mS-DLAS model. Collectively, 71% of the auto-segmented contours by the three models had relatively high quality. Additionally, the obtained mS-DLAS successfully segmented 9 out of 16 MRI sequences that were not used in the model training.

**Conclusion:**

We have developed an MRI-based mS-DLAS model for auto-segmenting of upper abdominal organs on MRI. Multi-sequence segmentation is desirable in routine clinical practice of RT for accurate organ and target delineation, particularly for abdominal tumors. Our work will act as a stepping stone for acquiring fast and accurate segmentation on multi-contrast MRI and make way for MR only guided radiation therapy.

## 1 Introduction

Magnetic resonance images (MRIs) offering both anatomic and functional information along with superior soft tissue contrast are becoming a leading image modality for radiation therapy (RT) planning and delivery (1, 2). For certain tumor sites, such as abdomen, MRI is the image of choice for accurate definition of the targets and/or organs at risk (OARs) (3). In MRI-based RT simulation, MRIs of multi-sequences with varying contrast, slice thickness, pixel size, pulse times, and other parameters are often acquired allowing to optimally define tumors and/or OARs (4). For upper abdominal anatomy, where soft high tissue contrast is essential, multi-sequence MRIs are desirable for the delineation (5, 6). For example, it’s a common practice to use T1 weighted MRI to define pancreas tumor and T2 weighted MRI to delineate OARs (e.g., duodenum) (4). While useful, it is not practical to manually segment all acquired images, hence a common practice is either to use and segment a single sequence for planning in the absence of contour availability on multiple sequences or use CT-MRI registration to take advantage of MRI information when segmenting CT. However, this in itself is a time-consuming practice and is riddled with CT-MRI registration uncertainties. To take full advantage of information from multiple MRI sequences and to improve organs at risk (OARs) and tumor segmentation, a fully automated solution is desirable.

Big data driven, deep learning auto-segmentation (DLAS) has shown great potential and success for RT planning and delivery guidance for a large cohort of tumor sites (7–9). However considerably less previous work is seen for DLAS in abdomen, especially on MRI, due in part to the complexity of this site in regard to the huge variability of shape and volume of the digestive organs (e.g., stomach, duodenum, bowels) and regularly occurring motion and intensity artifacts in MRI (10). Additionally, most of previous works were focused on the training and/or testing of DLAS models based on single MRI sequence. For example, Fu et al. proposed a CNN based prediction-correction network, with embedded dense block for auto-segmentation of 6 abdominal organs on a single sequence TrueFISP MRI. The novelty and high accuracy of DLAS was achieved by introducing a sub-CNN correction network in conjunction with the original prediction algorithm (11). Liang et al. used TruFISP MRI for training and T1 MRI for testing to auto-segment 5 organs using a fused approach, incorporating MRI features and a self-adaptive, active learning classification algorithm (12). BoBo et al. used a classical fully convolutional neural network (FCNN) to auto-segment 6 organs in the abdomen (13). Chen et al. used two-dimensional U-net and a densely connected network to segment 10 organs on T1 VIBE MRI (14). Zhao et al. reported a novel multi-scale segmentation network MTBNet, (multi-to-binary block) integrated with the ProbGate and an auxiliary loss to segment 4 organs on T1-DUAL in phase and T2-SPIR MRIs, respectively (15). Jiang et al, adopted a more unique approach of using CT labels to segment unlabeled T1 and T2 MRIs. They used a variational auto-encoder to segment 4 large to medium sized organs in abdomen (16). Li et al, developed patient specific auto-segmentation model using single sequence daily MRI (T2 Haste) (17). To our knowledge, there is no study so far reporting single DLAS model for abdominal organs based on multi-sequence MRIs.

To improve efficiency in MRI-based RT, it is desirable to develop single DLAS model for multi-sequence MRIs. The aim of this work was to develop a generic multi-sequence DLAS model for 12 common abdominal organs based on 4 types of commonly used MRI sequences in RT simulation. In addition, two sequence specific DLAS models were also developed based on the same training and testing datasets. The performance of the 3 DLAS models were evaluated based on their clinical applicability, e.g., accuracy of the auto-segmented contours, labor- and timesaving of segmentation when compared to manual contouring.

## 2 Materials and methods

### 2.1 MRI datasets

**Figure 1:**
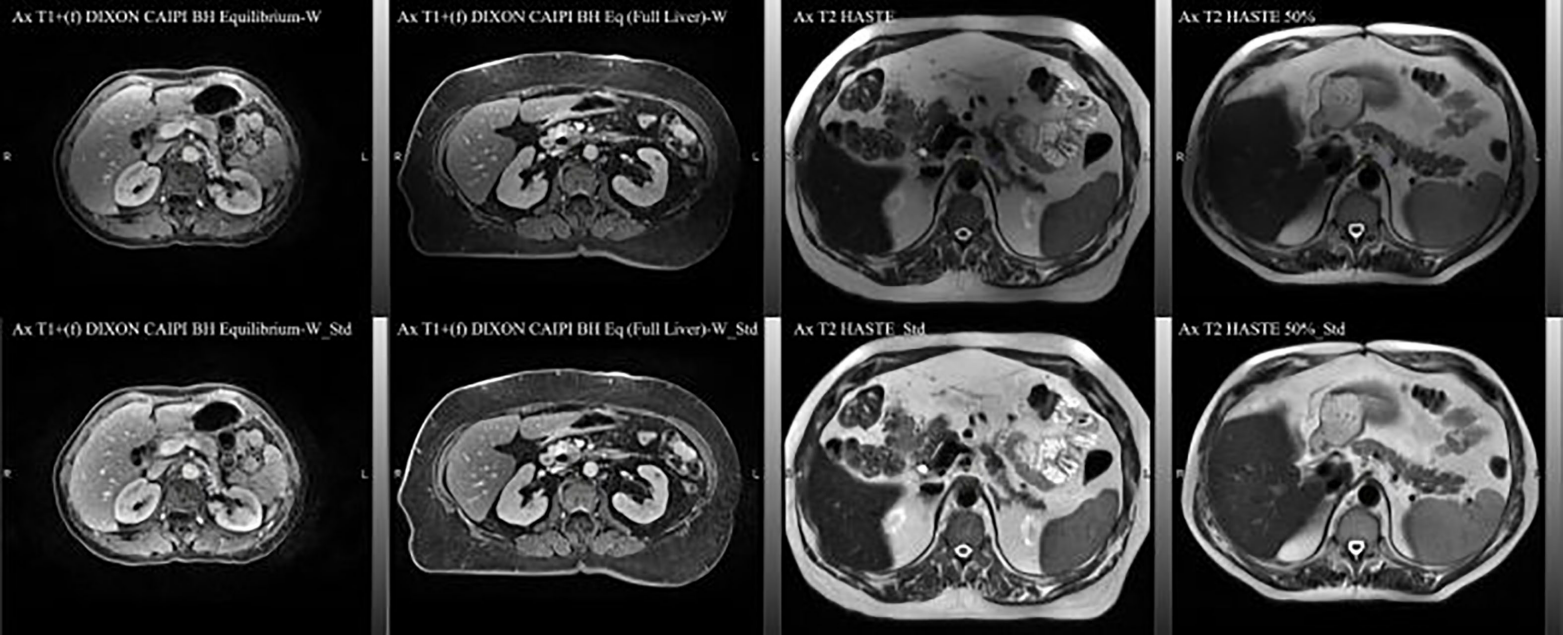
Representative axial slices of the four image sequences used in the DLAS training and testing. Top) raw images; bottom) pre-processed, standardized images.

The MRI data acquired during routine RT simulation of 71 patients with abdominal tumors, each with 2 out of the 4 MRI sequences, two post-contrast T1 (Ax T1+(f) DIXON CAIPI BH Equilibrium W, Ax T1+(f) DIXON CAIPI BH Eq (Full Liver)_W) and two motion-triggered T2 (Ax T2 half-Fourier single-shot turbo spin-echo (HASTE) and AX T2 HASTE 50%) sequences from a 3T MRI simulator (Verio, Siemens) were used for the DLAS training (61 patients = 121 datasets) and testing (10 patients = 20 datasets). The image acquisition parameters for these sequences are summarized in **Table 1**. As the imaging data comprised of a wide range of contrast variations and field-of-view settings, all images were pre-processed using an in-house standardization workflow including: 1) bias field correction, using an N4 algorithm (18), 2) noise filtering using anisotropic diffusion (19), and 3) intensity normalization by thresholding to volumetric median. Representative slices of the 4 image sequences before and after the standardization are shown in **Figure 1**. For training, a Z-score normalization method was used on both T1 and T2 weighted images for each patient base to better accommodate the variations in the multi-sequence images. To avoid negative values of outlier pixels, the pixel percentage intensity distribution of [0.25%-99.75%] was used to calculate the mean and standard deviation that were used subsequently to normalize the whole image.

**Table 1:** Image acquisition parameters of the T1 and T2 imaging sequences.

Sequence Parameters	T1+(f) DIXON CAIPI BH (Equilibrium/Eq (Full Liver))-W	T2 (HASTE/HASTE 50%)
MR acquisition type	3D	2D
Repetition time (TR)	4.29 msec	2000 msec
Echo time (TE)	1.23 msec	98 msec/96 msec
Slice thickness	3 mm	3 mm/5mm
Pixel spacing	[1.640625, 1.640625] mm	[1.1875, 1.1875] mm
Resolution	3 mm	3 mm/5mm
Flip angle	9°	150°

Contours of the 12 organs of interest: aorta, large and small bowel, duodenum, esophagus, left and right kidney, liver, pancreas, spinal cord, spleen, and stomach, were either created or slice-by-slice reviewed by a single researcher (trained by experienced oncologists) using oncologist provided and followed guidelines and references (20, 21). Two experienced radiation oncologists specialized for abdominal tumors, verified/spot check the final contours for accuracy and consistency in contouring. Manual contouring was done using a commercial clinical contouring tool (MIM version 6.8.6, MIM Software Inc., Beachwood, OH, USA). These manual contours were considered as manual reference contours (MRC) in the DLAS training and testing.

### 2.2 Deep learning based auto-segmentation (DLAS)

A modified ResUnet3D network was used to develop the MRI based auto-segmentation models. The details of the algorithm were reported in our previous work (22). The algorithm used encoding and decoding structures to learn and generate label maps. Short- and long-range connections were introduced in the convolutional residual blocks, to among other things, decrease the number of iterations, improve information transmission, and preserve integrity of high-resolution features. In this work, a few additional considerations were implemented. Two data argumentation techniques were adopted to create additional training data with larger variations to improve the model robustness and to avoid the potential overfitting. Initially, an in-house developed 3D elastic transformation with a minor random deformation on both images and labels was applied on the fly for each case. These data can potentially accelerate DLAS model training by learning embedding features, as opposed to the memorization of the pixel location of the organs. The second data argumentation method employed a gamma intensity transformation with a possibility p=0.3, with a gamma uniform distribution of [0.7, 1.3]. This allowed us to mimic some level of intensity variation across different MRIs (23). A common practice in DLAS development is to crop the images, limiting information to the relevant regions or organs of interest, thus reducing the demand for large memory. In this work, we used original image size as input to preserve and take advantage of the relative spatial localization constraints for the multiple organs of interest. The final presented models were trained with the original image input size of 320 × 320 × 32 pixels. Moreover, a series of tests were conducted including 5-fold cross validation and the impacts of spatial resolution and scan length along z-axis, in order to optimize DLAS performance for small, multi-segmented organs like pancreas and duodenum. Three DLAS models were developed: 1) a multi-sequence model (mS-DLAS), trained using 4 MRI sequences, 2) a T1 model (T1-M), trained using the MRI data of the two T1 sequences, 3) a T2 model (T2-M), trained with the MRIs of the two T2 sequences.

### 2.3 Performance of auto-segmentation models

Performances of the obtained T1-M and T2-M models were evaluated using the T1 and T2 images of the 10 testing cases, respectively, whereas both these T1 and T2 images (total of 20 datasets) were used for the mS-DLAS testing. The following quantitative metrics were calculated by comparing the auto-segmented to the manual reference contours:

1. DSC to measure volumetric overlap.
2. Mean distance to agreement (MDA in mm) to measure mean distance between points on each contour set.
3. 95 percentile Hausdorff distance (HD95% in mm) to achieve 95 percentiles of the maximum distance between points on each contour set (24).
4. Percent volume difference (PVD) to account for difference between the two volumes, a negative PVD indicates an under drawn auto-segmented contour and vice versa (25).
5. Surface DSC (sDSC) to measure the overlap of two surfaces (26). Unlike the volume overlap, a boundary overlap is expected to provide more information and higher accuracy, essentially since segmentation is an organ boundary identification process.
6. Added path length (APL/mm) is the path length from the manual reference contour that had to be added to correct the auto-segmented contour boundary (surface) (27, 28). Instead of standard APL, here we introduce a relative APL (rAPL in mm/cc), which is defined as APL divided by the volume. The rationale of using rAPL is to calculate the editing distance per organ independent of the organ volume. Otherwise, it can lead to a large APL value, just because the organ volume is large and not necessarily because it needs large edits.

Per our clinical experience and in-house discussion based on literature survey, for sDSC and APL calculation, a tolerance of 2 mm was used. This tolerance is an estimation of the clinically expected inter-observer variations in manual segmentation.

**Figure 2:**
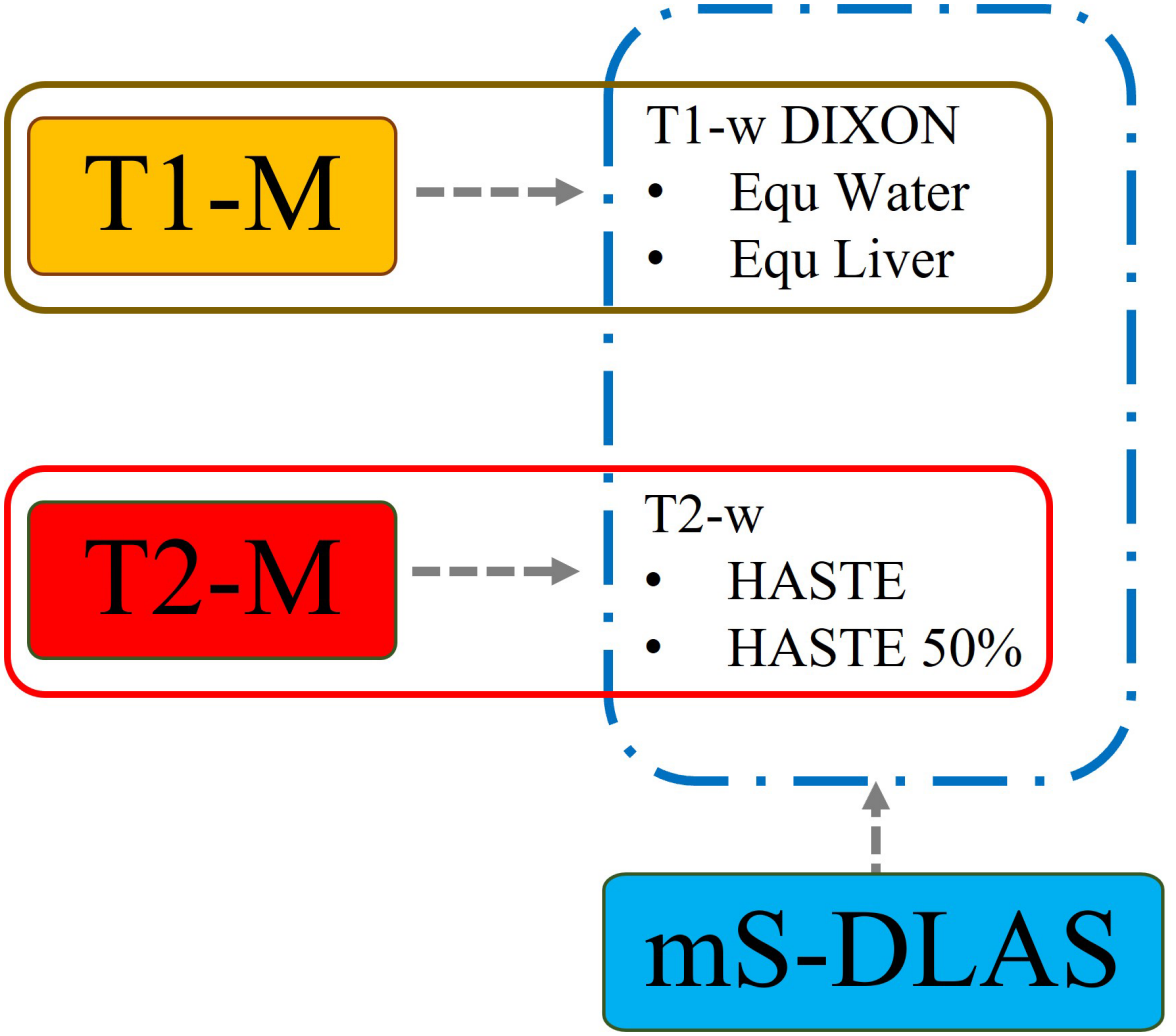
Schematic of DLAS models implemented to respective MRI sequences.

Based on the above metrics, the performance of the mS-DLAS on the MRIs of the 4 sequences was compared with T1-M on T1 images and with T2-M on T2 images using the testing datasets of the same sequences as those used in the model training. A schematic of the testing data used for each model is shown in (**Figure 2**). In addition, to test the model robustness, the mS-DLAS model was applied to randomly selected 5 MRI datasets acquired using 16 sequences different from the 4 sequences used in the model training. These different sequences included water and fat suppressed protocols, arterial and venous imaging post contrast, delayed, and in/out-of-phase.

## 3 Results

**Figure 3:**
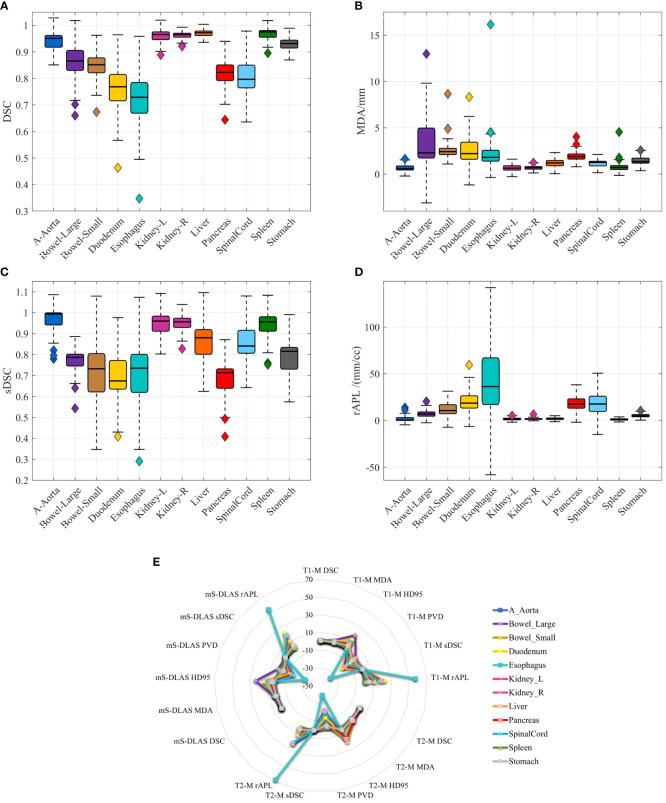
**(A–D)** Boxplots of 4 accuracy metrics calculated for the auto-segmented contours created by the mS-DLAS model for 12 organs. High DSC and sDSC values and consequently low MDA and rAPL trends are observed for most of the organs, thus indicating accuracy of DLAS; **(E)** A radar plot of average values of each metric for each of the 12 organs and each of the 3 developed models. The radii or spoke, as represented by the solid line length is the average value of a metric per organ, relative to the maximum value of the metric across all the 12 organs and the 3 models.

**Figure 4:**
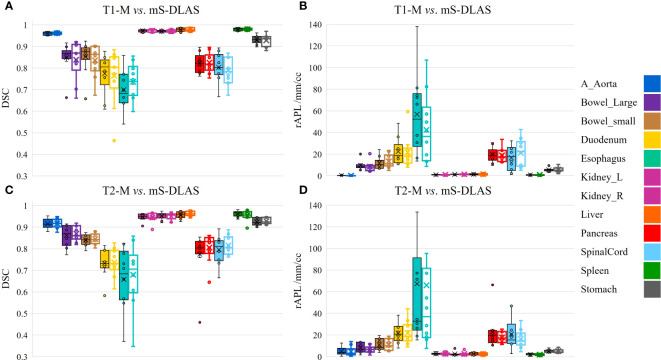
Organ based performance of T1 model *vs*. mS-DLAS on T1 MRI and T2 model *vs*. mS-DLAS on T2 MRI. Empty box plots represent the mS-DLAS, the filled box plots represent T1-M and T2-M.

Segmentation times per case for the 12 organs using one of the three generated models were observed to be in the range of 11-21 seconds, with an average of 15 seconds, on a common computer hardware (Intel Xeon Gold, NVIDA GeForce RTX 2080 Ti and NVIDA Quardro P2000 @ 2.6 GHz 128 GB RAM). **Figures 3A-D** presents boxplots of 2 commonly used metrics DSC, MDA and 2 newly introduced sDSC, and rAPL metrics calculated from the auto-segmented contours by the mS-DLAS model for the 12 organs on the 20 testing datasets of the same 4 sequences as in the training datasets. **Figure 3E** presents a radar plot of average values of each metric for each of the 12 organs and each of the 3 developed models. The radii or spoke, as represented by the solid line length is the average value of a metric per organ, relative to the maximum value of the metric across all the 12 organs and the 3 models. For example, the DLAS performed poorly for the esophagus as indicated by the long spoke. Whereas relative short spokes with similar patterns were seen for rest of the organs for the 3 models. The average values of DSC over all the organs on the testing datasets of the same sequences as in the model training were 0.882, 0.860 and 0.874, for the T1-M, T2-M and mS-DLAS, respectively. The corresponding values of MDA were 1.573, 2.128 and 1.790. The mS-DLAS outperformed the T2-M model on T2 MRIs, whereas the T1-M model outperformed the mS-DLAS on T1 MRIs, as shown in (**Table 2** and **Figure 4**).

**Table 2:** Comparison of single sequence models (T1-M and T2-M) with the general sequence independent model ms-DLAS on T1 and T2 images.

	DSC	MDA/mm	HD95%/mm	PVD/%	sDSC	rAPL_/_mm/cc
Model performance on T1 images						
T1-M	0.882	1.573	6.171	-8.516	0.844	12.18
mS-DLAS	0.881	1.637	7.078	-8.373	0.846	11.2
Model performance on T2 images						
T2-M	0.860	2.128	8.542	-10.71	0.777	14.25
mS-DLAS	0.867	1.942	7.777	-9.535	0.785	13.11

**Table 3** presents the average values of the quantitative accuracy metrics calculated for the 12 organs for the 3 models across the testing cases. The numbers highlighted in bold in **Table 3**, represent the highest accuracy observed per organ, across all the models. Moreover, each accuracy metric output was divided into 3 categories, as shown in **Table 3**: 1) “best” (pink filled), 2) “good” (green filled), indicating need for minor adjustments, and 3) “sub-optimal” (no fill), indicating the need for modifications. The “best” and “good” accuracy thresholds were dictated as follows: DSC ≥ 0.9, MDA ≤ 1.5mm, HD95% ≤ 5mm, PVD ≤ 3%, SDSC ≥ 0.85, rAPL ≤ 5mm/cc; and DSC ≥ 0.8, MDA ≤ 3mm, HD95% ≤ 10mm, PVD ≤ 6%, SDSC ≥ 0.75, rAPL ≤ 10mm/cc, respectively. It was observed that 71% of the auto-segmented contours by the 3 models for all organs fall into the best and good categories.

**Table 3:** The 3D average values of 6 contour accuracy metrics calculated for the auto-segmented contours for 12 organs using 3 DLAS models.

	DSC			MDA/mm			HD95%/mm			PVD/%			sDSC			rAPL_/_mm/cc		
	T1-M	T2-M	mS-DLAS	T1-M	T2-M	mS-DLAS	T1-M	T2-M	mS-DLAS	T1-M	T2-M	mS-DLAS	T1-M	T2-M	mS-DLAS	T1-M	T2-M	mS-DLAS
A_Aorta	**0.96**	0.91	0.94	**0.5**	1.05	0.75	**1.62**	2.64	2.11	**-2.15**	-8.95	-5.71	**0.99**	0.89	0.95	**0.13**	5	2.31
Bowel_Large	0.85	**0.86**	0.85	3.8	**2.85**	3.85	18.97	**14.87**	23.68	**0.27**	-4.51	1.73	0.73	**0.77**	0.76	9.54	7.9	**7.54**
Bowel_Small	**0.85**	0.84	0.84	**2.38**	3.42	2.81	**9.39**	19.52	14.51	-7.77	**-6.06**	-11.8	**0.74**	0.68	0.71	11.54	**10.92**	12.07
Duodenum	**0.77**	0.73	0.75	**2.76**	3.01	2.92	**12.68**	12.69	**12.68**	-18.8	-13.8	**-13.5**	**0.72**	0.64	0.68	22.34	**21.65**	21.79
Esophagus	0.7	0.66	**0.71**	**2.26**	4	2.8	**8.87**	13.63	10.02	-36.3	-39.4	**-31.2**	0.7	0.62	0.7	56.79	67.31	**54.16**
Kidney_L	**0.97**	0.95	0.96	**0.51**	0.9	0.69	**1.62**	2.92	2.12	-1.9	**-0.83**	-2.91	**0.98**	0.91	0.95	**0.96**	2.66	1.75
Kidney_R	**0.97**	0.95	0.96	**0.55**	0.84	0.71	**1.69**	2.6	2.1	**-0.06**	-2.95	-2.64	**0.97**	0.92	0.94	**1.35**	2.45	1.85
Liver	**0.98**	0.96	0.97	**0.99**	1.63	1.24	**2.93**	5.64	3.92	**-1.64**	-3.74	-2.22	**0.91**	0.79	0.86	**0.139**	2.62	1.92
Pancreas	**0.82**	0.77	0.81	**1.92**	3.97	2.01	6.8	16.61	**6.57**	-16	-21.7	**-14.8**	**0.73**	0.63	0.68	19.3	22.8	**18.24**
SpinalCord	0.8	0.8	0.8	**1.2**	1.23	1.21	**3.01**	3.03	3.18	**-12**	-19.7	-17.8	**0.87**	0.82	0.85	**16.54**	20.86	18.74
Spleen	**0.98**	0.96	0.97	**0.57**	1.01	0.97	**1.8**	3.17	3.86	-1.97	-2.73	**-1.22**	**0.97**	0.9	0.93	0.721	1.99	1.26
Stomach	**0.93**	0.92	**0.93**	**1.44**	1.62	1.5	4.66	5.19	4.66	**-3.95**	-4.21	-5.37	**0.81**	0.76	0.79	5.45	**4.85**	5.38

Accuracy per metric is categorized as “best”, “good”, and “suboptimal” with pink, green and unfilled cells respectively. Bold numbers represent highest accuracy per metric for each organ, amongst the 3 models.

**Figure 5:**
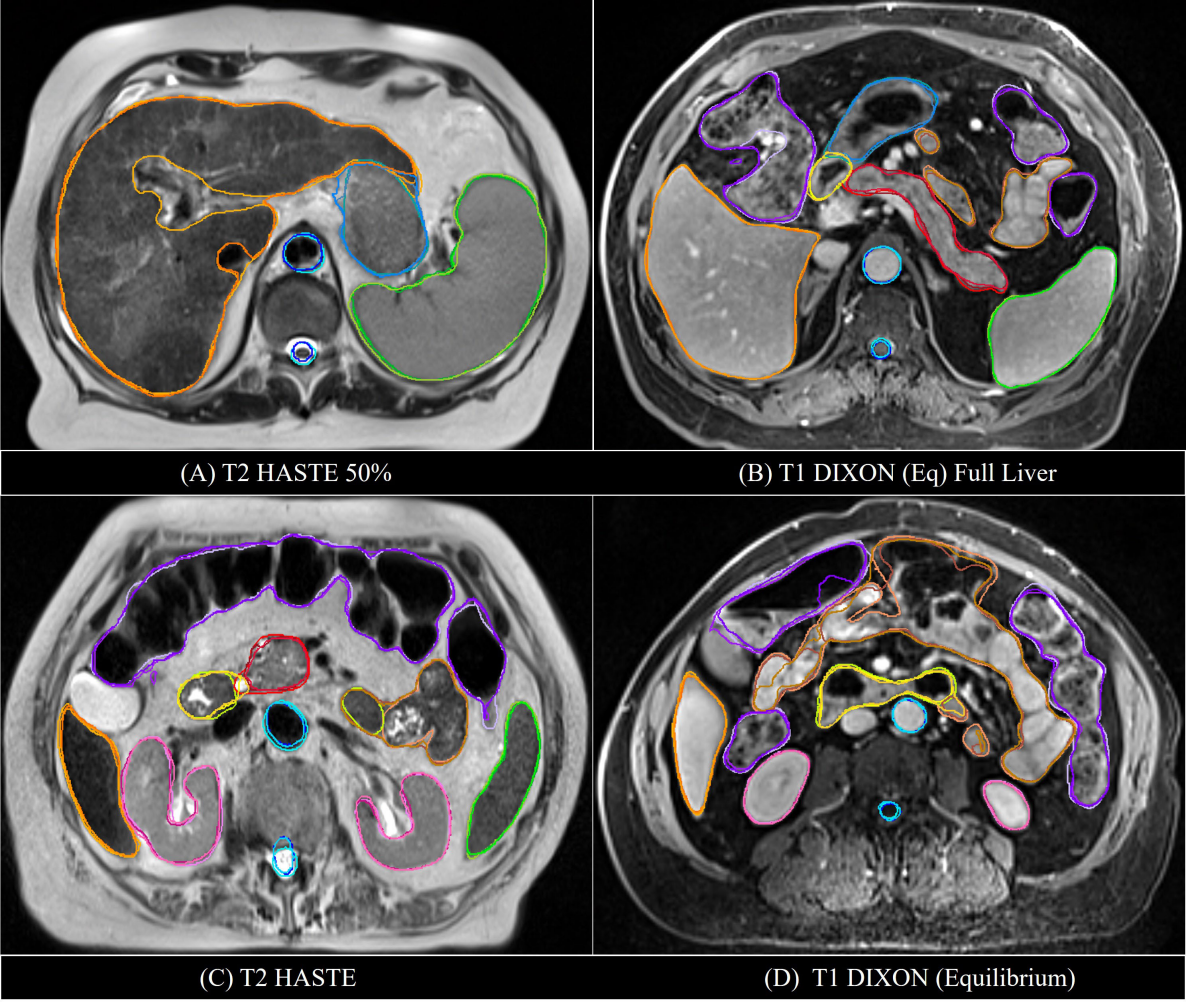
Comparison of the manual reference contours (light-colored line) with the auto-segmented contours by the multi-sequence mS-DLAS (dark-colored lines) and the sequence-specific models (e.g., T1-M or T2-M) (medium-colored lines) on four representative axial slices of 2 T2 **(A, C)** and 2 T1 **(B, D)** images.

Examples of mS-DLAS generated contours (dark-colored lines) on representative axial images of the 4 sequences are shown in **Figure 5**. On each image, the MRC (light-colored lines), and the auto-segmentation of the sequence-specific model corresponding to the image sequence (medium-colored lines) is also shown for comparison. As presented in **Table 3**, a reasonable overlap of auto-segmentations of the three DLAS model with the MRC was seen for majority of the organs. For all 3 models, the best performance was seen either for large/medium size organs with limited motion, or for organs with stable spatial dimensions e.g., liver, kidneys, spleen, stomach, and aorta. For long thin organs like esophagus, or multi-segment organs like pancreas and duodenum somewhat suboptimal segmentation accuracy was observed. The accuracy of all the 3 DLAS models was relatively inferior in the regions with organ abutting or junction (e.g., gastroduodenal junction, duodenojejunal flexure) as shown in **Figure 5C**.

As mentioned in the results section, 71% of the contours were found to be acceptable, however, to fix the “good” and “sub-optimal” DLAS contours, manual editing will be required. Hence, to facilitate and identify the extent and quality of the edits required the DLAS segmentation from the T1-M and T2-M models, an organ-based scorecard to categorically label a DLAS contour in terms of its editing functions and number of slices requiring edits was created. The score ranged from 1 to 6, with score 1 requiring no edits, corresponding to “best” category in **Table 3**, and score 6 requiring the re-creation of contour manually. The details of the scoring criteria and corresponding score of each organ are shown in the **Table 4**. Moreover, it was observed that, on average, the total editing time for the auto-segmented contours of the 12 organs in a case with average score of 3 (corresponding to “good” category in **Table 3**) was approximately 15 minutes, at least 25 minutes shorter than the manual contouring time.

**Table 4:** (A) Scorecard criteria (1-6) quantifying the extent of edits required for the auto-segmentation of T1-M and T2-M models. (B, C) Scorecard for the auto-segmentation on T1-images and T2 images.

(A)	# of edited slices	Editing time	Scoring Criteria	Organs involved
1	0	0	No edits required	Aorta, kidneys, spinalcord, spleen
2	< 3 or < 5	3 min	Deletion of extra inaccurate regions, expansion of whole organ like spinalcord	Kidneys, spleen, spinalcord
3	< 10	8 min	Fix incomplete segmentation of organs,	Duodenum, pancreas
4	20 or < 50%		Edits performed at organ junctions or hilum	Liver, kidneys
5	> 50%			Bowels, duodenum, esophagus
6	> 80%		It is recommended to redo the contour	Bowels

**Table:** 

(B)	A	D	E	K_L	K_R	L	P	SC	S	ST	B_L	B_S
1	2	5	2	2	2	2	5	3	2	2	5	5
2	1	5	5	2	1	4	3	2	2	4	5	6
3	3	4	2	2	2	2	3	1	2	2	4	4
4	2	3	5		3	3	4	1	2	3	6	5
5	1	5	2	2	2	2	3	1	2	3	5	5

**Table:** 

(C)	A	D	E	K_L	K_R	L	P	SC	S	ST	B_L	B_S
1	3	6	6	3	3	5	5	3	3	5	5	6
2	1	3	2	2	2	3	3	2	2	3	4	6
3	4	5	2	2	1	4	3	1	2	4	5	5
4	2	3	3		2	3	3	1	4	4	5	6
5	1	5	2	2	2	2	3	2	3	2	5	6

Each column with initials is the 12 organs (**A**orta, **D**uodenum, **K_R** (left kidney), **K_R** right kidney, **L**iver, **P**ancreas, **SC** (SpinalCord), **S**pleen, **ST**omach, **B_L** (large bowel), **B_S** (small bowel). Each row represents a testing case. Per Table A, each color represents a number on the score card, for example, pink cell represent score of 0, i.e., no edits required category.

**Figure 6:**
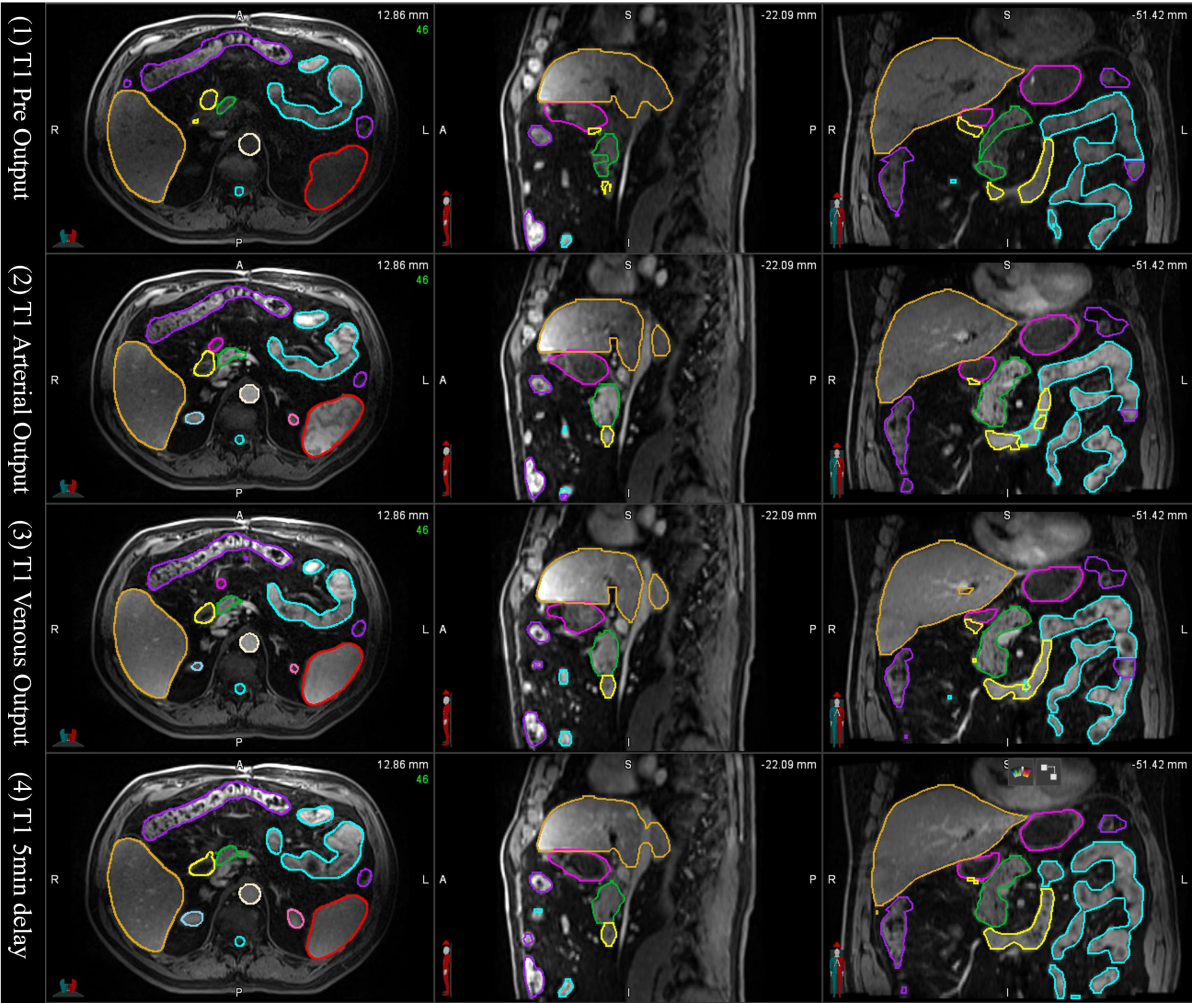
Auto-segmented contours by the multi-sequence mS-DLAS model on representative axial, sagittal and coronal images of 4 T1 weighted MRI sequences not used in the model training.

In the tests of applying the mS-DLAS model to the 16 different sequences not used in the training datasets, it was observed that the MS-DLAS model was able to create reasonable contours on the MRIs of 9 out of the 16 sequences. As an example, **Figure 6** shows the mS-DLAS segmentation on representative axial, sagittal and coronal images of four T1 sequences different to those used in the training. For the images with distinct contrast (e.g., fat suppressed, in-phase and out-of-phase), the mS-DLAS performed relatively poor, indicating that these images may need to be included in the model training.

## 4 Discussion

As multi-sequence MRIs are commonly used in RT, sequence independent DLAS solution is practically desirable in MRI-based RT. In this work, we developed such a general multi-sequence model (mS-DLAS) for abdominal organs and showed that the obtained mS-DLAS model generated contours of high quality and accuracy, as was expected from the 2 sequence-specific models (T1-M and T2-M) for the 2 sequences used in the model training. All models obtained were able to segment the 12 common organs on an MRI image in an average of 15 seconds. Considering an average of 75 slices in an MRI dataset, the auto-segmentation time of 0.2 sec/slice on MRI is less than 0.3 sec/slice on CT (total 70 seconds) as previously reported (22). The MS-DLAS model can even create reasonable contours on certain sequences that were not included in the model training datasets if the MRI contrasts are not substantially different from those in the training.

It has been discussed in the literature that the use of traditional contour accuracy metrics like DSC and MDA along with the acceptability criteria recommended by the TG 132 (27) is not sufficient to comprehensively evaluate DLAS performance from a practical point of view (28). To address this issue, we presented 6 volumetric and surface metrics to probe the multifaceted accuracy of the DLAS outputs. For example, of interest is PVD > 0 observed for large bowel with T1-model and the ms-DLAS model. This observation is a manifestation of the T1-w image contrast. Because of small contrast differences between the background and air in the bowels, DLAS often overestimated the segmentation. Large -ve PVD values, indicate under-drawn or incomplete segmentation, an expected observation for small organs like spinalcord or organs like pancreas and duodenum having multiple spatially located segments (tail, body, head for pancreas and junctions with stomach and ileum for duodenum) as shown in **Figures 3**, **5**. It has been reported that among the quantitative metrics, APL has the best correlation with the editing time taken to correct for the DLAS contour (29). Based on the evaluation using the 6 metrics, we observed that the T1-M model performed the best among the three models with average DSC, MDA, HD95%, PVD, SDSC, and rAPL values over all 12 organs of 0.882, 1.573, 6.171, -8.516, 0.844, and 12.18, respectively. The mS-DLAS performance was comparable to that for the two sequence-specific models with overall accuracy difference within the error ranges of the T1-M and T2-M results.

**Figure 7:**
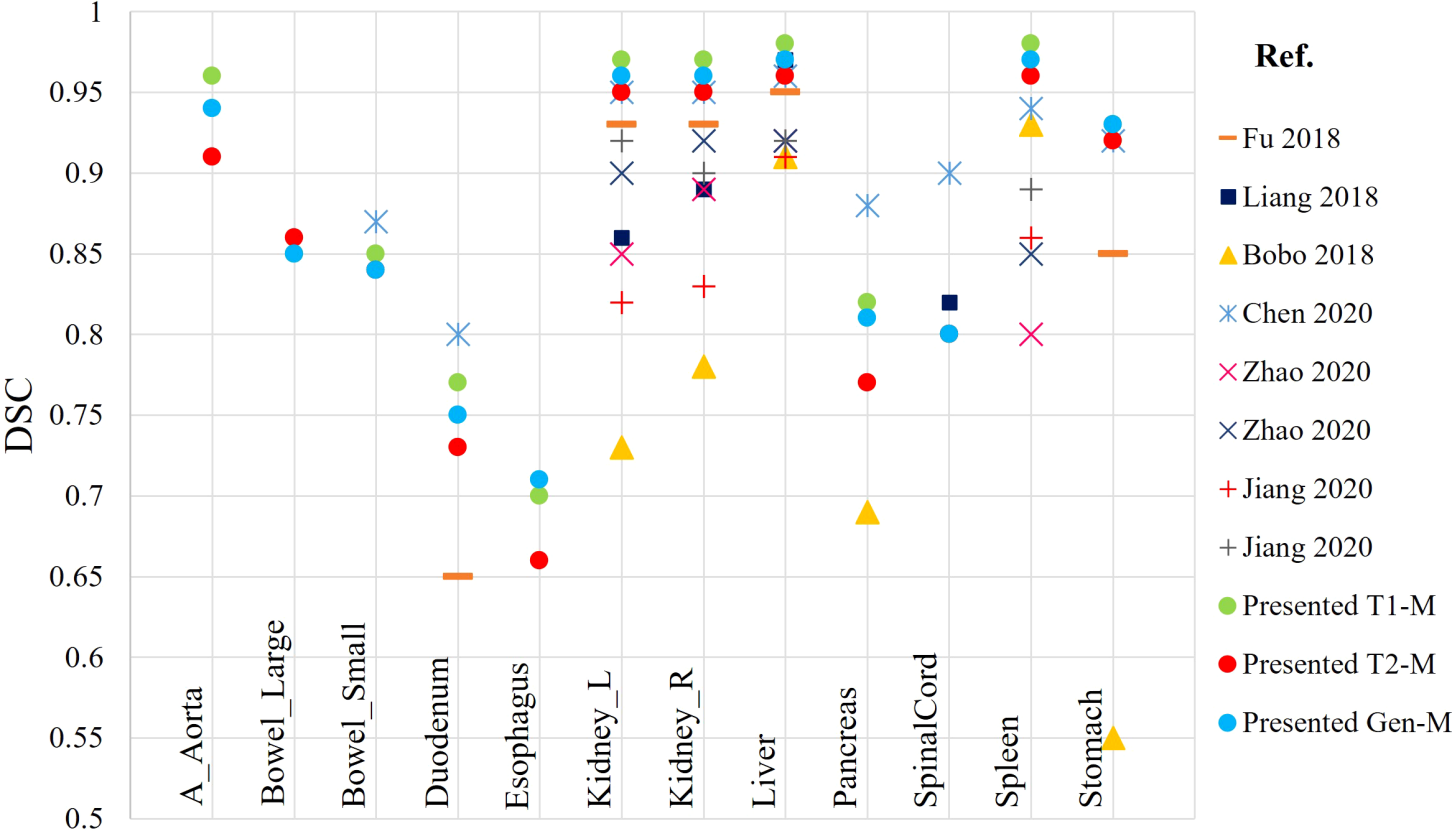
Comparison of DSC calculated from the presented DLAS models with previously reported auto-segmentation studies based on abdominal MRIs.

At the present time, development of DLAS on abdominal MRIs is generally sparse. Studies available in the literature are based on either CT or single sequence MRI. The present effort, reporting DLAS based on multi-sequence MRI, is the first of its kind. We compared the performance of the presently developed models with models reported in literature, trained on single MRI sequence, using different algorithms and training datasets (11–15). **Figure 7** reports the DSC values of the presented models as well as the studies existing in literature. Note that only DSC values of 9 organs were available from the previous works for the comparison. While no direct comparison of model performance was conducted, our DLAS models resulted in the highest DSC values (> 0.9) for five organs, i.e., kidneys, liver, spleen, and stomach, whereas comparable performance was observed for small bowel with DSC of 0.85 from the present work versus 0.87 reported by Chen et al. (14), and for duodenum and pancreas with DSC of 0.77 and 0.82 from this work versus 0.8 and 0.88 in the Chen’s work (14).

A concern in MRI-based DLAS is the intensity/signal distortions in the MRI. While various pre-processing techniques were used in this study to minimize this effect, high intensity inhomogeneity or significant drop in signal can lead to inaccurate auto-segmentation. It was observed that the best DLAS occurred on images with clear and high contrast (the histogram had two clear, sharp peaks), whereas relatively poor performance was seen in images with poor contrast and intensity, e.g., at the superior edge of the upper abdomen region that led to inaccurate auto-segmentation of liver and stomach in the superior slices of the scan. Another such example is esophagus, as shown by large variations in accuracy metrics (**Figure 3**). Bad segmentation of esophagus in this case is associated to missing DLAS, because of poor image contrast, leading to inaccurate boundary distinction. Moreover, poor DLAS results were observed in the cases where organs were removed or substantially deformed due to surgical procedures (e.g., pancreaticoduodenectomy) performed before RT. In such situations, which happen to be a small cohort of patients in our clinic, additional caution needs to be exercised for using DLAS as it can lead to inaccurate or even erroneous contours for the organs near the missing or deformed organs.

For MRI-based mS-DLAS to be used in routine clinical practice of RT for abdominal tumors, substantial future work is required to develop robust global DLAS models where large datasets of multi-machine and multi-sequence MRIs are used for the model training and testing.

## 5 Conclusions

We developed a multi-sequence deep learning auto-segmentation model based on abdominal MRIs. The proposed model can learn and segment on multi-contrast T1, T2 MRI images, included in its training and on sequences not used in training but are used in routine clinical practices, for example venous and arterial scans. The mS-DLAS was found to be fast and accurate for most of the organs, using 6 accuracy metrics and a scorecard criterion to predict editing times of these DLAS contours. For MR only RT and MRgART acquisition of new sequences to facilitate planning and treatment is part of the clinical processes, and integration of sequence specific DLAS models in clinical workflow will become a labor-intensive task, as it will require updates based on clinical needs. Our work to develop single DLAS model to segment multi-sequence, multi-contrast MRI is a potential solution to facilitate this issue. For future studies, we aim to improve the sequence-independent aspect, by training a global DLAS model, to incorporate multi-machine MRIs, that are desirable in routine clinical practice of RT, particularly for MR guided adaptive treatments of abdominal tumors.

## Data availability statement

Research data are stored in an institutional repository and selective data will be shared upon request to the corresponding authors.

## Ethics statement

The studies involving human participants were reviewed and approved by IRB. Written informed consent for participation was not required for this study in accordance with the national legislation and the institutional requirements.

## Author contributions

AA carried out the study, including data acquisition, qualitative analysis, and manuscript writing. JX and DT developed and integrated the auto-segmentation models. YZ and JD developed the accuracy metrics code. WH and BE checked the manual reference contours training and testing data. EP helped data acquisition. XL designed and oversaw the study and finalized the manuscript. All authors contributed to manuscript revision, read, and approved the submitted version.

## References

[1] Jonsson J Nyholm T Söderkvist K. The rationale for MR-only treatment planning for external radiotherapy. Clin Trans Radiat Oncol (2019) 18:60–5. doi: 10.1016/j.ctro.2019.03.005 PMC6630106 31341977

[2] Devic S. MRI Simulation for radiotherapy treatment planning. Med Phys (2012) 39(11):6701–11. doi: 10.1118/1.4758068 23127064

[3] Glide-Hurst CK Paulson ES McGee K Tyagi N Hu Y Balter J. Task group 284 report: magnetic resonance imaging simulation in radiotherapy: considerations for clinical implementation, optimization, and quality assurance. Med Phys (2021) 48(7):e636–70. doi: 10.1002/mp.14695 PMC8761371 33386620

[4] Paulson ES Erickson B Schultz C Li XA. Comprehensive MRI simulation methodology using a dedicated MRI scanner in radiation oncology for external beam radiation treatment planning. Med Phys (2015) 42:28–39. doi: 10.1118/1.4896096 25563245

[5] Heerkens HD Hall WA Li XA Knechtges P Dalah E Paulson ES. Recommendations for MRI-based contouring of gross tumor volume and organs at risk for radiation therapy of pancreatic cancer. Pract Radiat Oncol (2017) 7:126–36. doi: 10.1016/j.prro.2016.10.006 28089481

[6] Noel CE Zhu F Lee AY Yanle H Parikh PJ. Segmentation precision of abdominal anatomy for MRI-based radiotherapy. Med Dosim (2014) 39(3):212–7. doi: 10.1016/j.meddos.2014.02.003 PMC4211895 24726701

[7] Samarasinghe G Jameson M Vinod S Field M Dowling J Somya A. Deep learning for segmentation in radiation therapy planning: a review. J Med Imaging Radiat Oncol (2021) 65:578–95. doi: 10.1111/1754-9485.13286 34313006

[8] Harrison K Pullen H Welsh C Oktay O Alvarez-Valle J Jena R. Machine learning for auto-segmentation in radiotherapy planning. Clin Oncol (2022) 34:74–88. doi: 10.1016/j.clon.2021.12.003 34996682

[9] Chen X Wang X Zhang K Fung KM Thai TC Moore K. Recent advances and clinical applications of deep learning in medical image analysis. Med Image Anal (2022) 79:102444. doi: 10.1016/j.media.2022.102444 35472844 PMC9156578

[10] Cusumano D Boldrini L Dhont J Fiorino C Green O Güngör G. Artificial intelligence in magnetic resonance guided radiotherapy: medical and physical considerations on state of art and future perspectives. Physica Med (2021) 85:175–91. doi: 10.1016/j.ejmp.2021.05.010 34022660

[11] Fu Y Fu Y Mazur TR Wu X Liu S Chang X. A novel MRI segmentation method using CNN-based correction network for MRI-guided adaptive radiotherapy. Med Phys (2018) 45(11):5129–37. doi: 10.1002/mp.13221 30269345

[12] Liang F Qian P Su KH Baydoun A Leisser A Van Hendent S. Abdominal, multi-organ, auto-contouring method for online adaptive magnetic resonance guided radiotherapy: an intelligent, multi-level fusion approach. Artif Intell In Med (2018) 90:34–41. doi: 10.1016/j.artmed.2018.07.001 30054121

[13] Bobo MF Bao S Huo Y Yao Y Virostko J Plassard AJ. Fully convolutional neural networks improve abdominal organ segmentation. Proc SPIE Int Soc Opt Eng (2018) 10574:105742V. doi: 10.1117/12.2293751 PMC5992909 29887665

[14] Chen Y Ruan D Xiao J Wang L Sun B Saouaf R. Fully automated multiorgan segmentation in abdominal magnetic resonance imaging with deep neural networks. Med Phys (2020) 47(10):4971–82. doi: 10.1002/mp.14429 PMC7722015 32748401

[15] Zhao X Huang M Li L Qi XS Tan S. Multi-to-binary network (MTBNet) for automated multi-organ segmentation on multi-sequence abdominal MRI images multi-to-binary network. Phys Med Biol (2020) 65:165013. doi: 10.1088/1361-6560/ab9453 32428898

[16] Jiang J Veeraraghavan H. Unified cross-modality feature disentangler for unsupervised multi-domain MRI abdomen organs segmentation. Med Image Comput Comput Assist Interv (2020) 12262:347–58. doi: 10.1007/978-3-030-59713-9_34 PMC7757792 33364627

[17] Li Z Zhang W Li B Zhu J Peng Y Li C. Patient specific daily updated learning auto-segmentation for MRI-guided adaptive radiotherapy. Radiother Oncol (2022) 177:222–30. doi: 10.1016/j.radonc.2022.11.004 36375561

[18] Sled JG Zijdenbos AP Evans AC. A nonparametric method for automatic correction of intensity nonuniformity in MRI data. IEEE Trans Med Imag (1998) 17(1):87–97. doi: 10.1109/42.668698 9617910

[19] Perona P Malik J. Scale-space and edge detection using anisotropic diffusion. IEEE T Pattern Ana (1990) 12:629–39. doi: 10.1109/34.56205

[20] Jabbour SK A Hashem S Bosch W Kim TK Finkelstein ST Anderson BM. Upper abdominal normal organ contouring guidelines and atlas: a Radiation Therapy Oncology Group consensus. Pract Radiat Oncol (2014) 4(2):82–9. doi: 10.1016/j.prro.2013.06.004 PMC4285338 24890348

[21] Lukovic J Henke L Gani C Kim TK Stanescu T Hosni A. MRI-Based upper abdominal organs-at-Risk atlas for radiation oncology. Int J Radiat Oncol Biol Phys (2020) 106(4):743–53. doi: 10.1016/j.ijrobp.2019.12.003 31953061

[22] Amjad A Xu J Thill D Lawton C Hall W Musaddiq JA. General and custom deep learning auto-segmentation models for organs in head and neck, abdomen, and male pelvis. Med Phys (2022) 49(3):1686–700. 10.1002/mp.15507 PMC8917093 35094390

[23] Gonzalez R Woods R. Digital image processing. 4th ed. New York, NY: Pearson Publishers (2017).

[24] Sharp G Fritscher KD Pekar V Peroni M Shusharina N Veeraraghavan H. Vision 20/20: perspectives on automated image segmentation for radiotherapy. Med Phys (2014) 41:050902. doi: 10.1118/1.4871620 24784366 PMC4000389

[25] Schoemaker D Buss C Head K Sandman CA Davis EP Chakravarty MM. Hippocampus and amygdala volumes from magnetic resonance images in children: assessing accuracy of FreeSurfer and FSL against manual segmentation. Neuroimage (2016) 129:1–14. doi: 10.1016/j.neuroimage.2016.01.038 26824403 PMC7243960

[26] Nikolov S Blackwell S Zverovitch A Mendes R Livne N De Fauw J. Clinically applicable segmentation of head and neck anatomy for radiotherapy: deep learning algorithm development and validation study. J Med Internet Res (2021) 23(7):e26151. doi: 10.2196/26151 34255661 PMC8314151

[27] Brock KK Mutic S McNutt TR Li H Kessler ML. Use of image registration and fusion algorithms and techniques in radiotherapy: report of the AAPM radiation therapy committee task group no. 132. Med Phys (2017) 44(7):e43–76. doi: 10.1002/mp.12256 28376237

[28] Kiser KJ Braman A Stieb S Fuller CD Giancardo L. Novel autosegmentation spatial similarity metrics capture the time required to correct segmentations better than traditional metrics in a thoracic cavity segmentation workflow. J Digit Imaging (2021) 34(3):541–53. doi: 10.1007/s10278-021-00460-3 PMC8329111 34027588

[29] Vaassen F Hazelaar C Vaniqui A Gooding M van der Heyden B Canters R. Evaluation of measures for assessing time-saving of automatic organ-at-risk segmentation in radiotherapy. Phys Imaging Radiat Oncol (2020) 13:1–6. doi: 10.1016/j.phro.2019.12.001 33458300 PMC7807544

