# Supplementary material for: Deep learning auto-segmentation on multi-sequence magnetic resonance images for upper abdominal organs
Source: Front Oncol. 2023 Jul 6;13:1209558. doi: 10.3389/fonc.2023.1209558 (PMC10358771; doi:10.3389/fonc.2023.1209558)

An example of poor auto-segmentation observed for; A) image with poor contrast, and B) surgically absent organ.

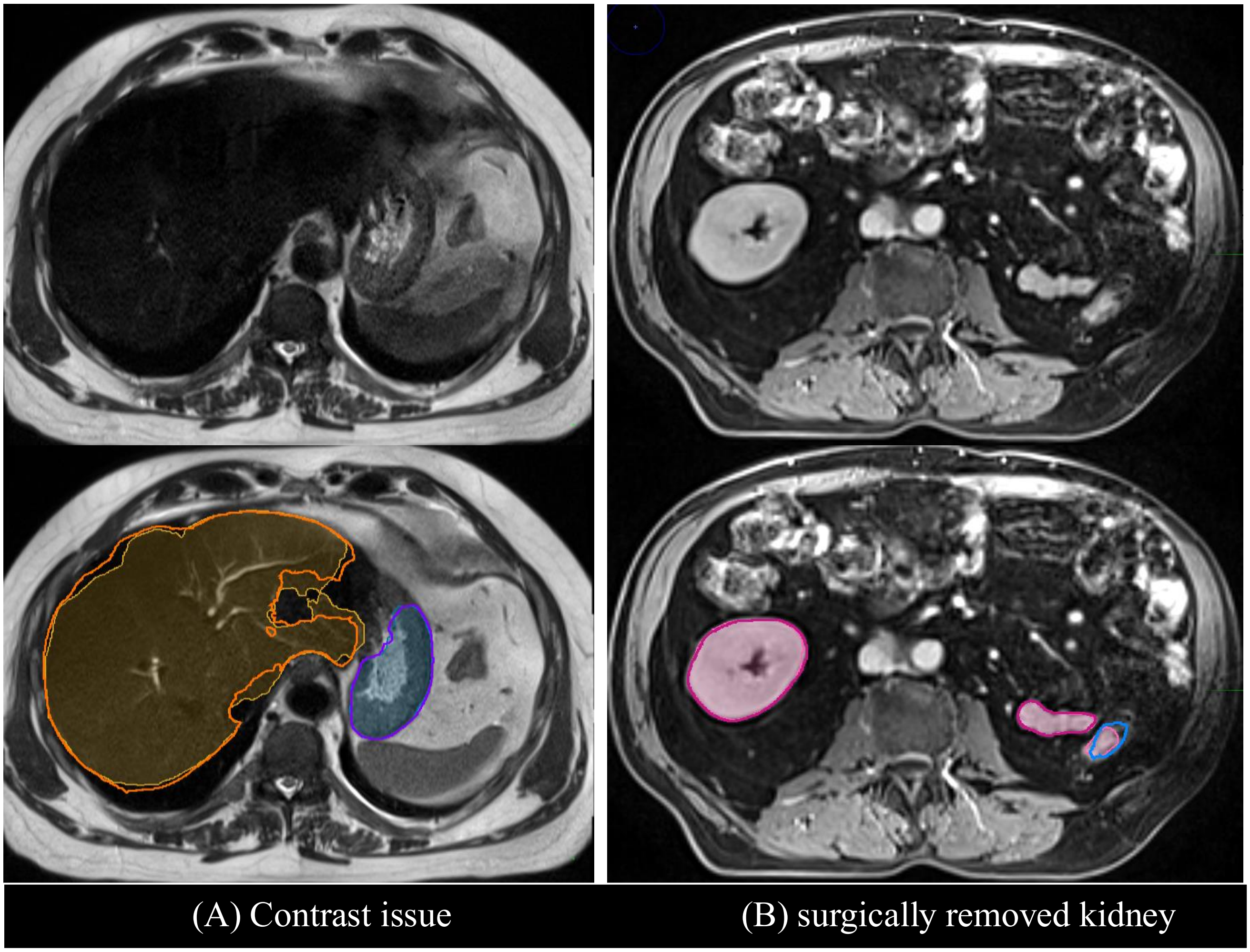

Supplement: Supplementary file 1 [file Image_1.pdf]
